# Supplementary material for: Vitamin D Modified DSS-Induced Colitis in Mice via STING Signaling Pathway
Source: Biology (Basel). 2025 Jun 18;14(6):715. doi: 10.3390/biology14060715 (PMC12190092; doi:10.3390/biology14060715)
Supplement: Supplementary file 1 [file biology-14-00715-s001.zip › Supplementary Materials S1: 16s-report of gut microbiota/Groups/diff_analysis/TukeyHSD/Tukey_KEGG_rotate.pdf]

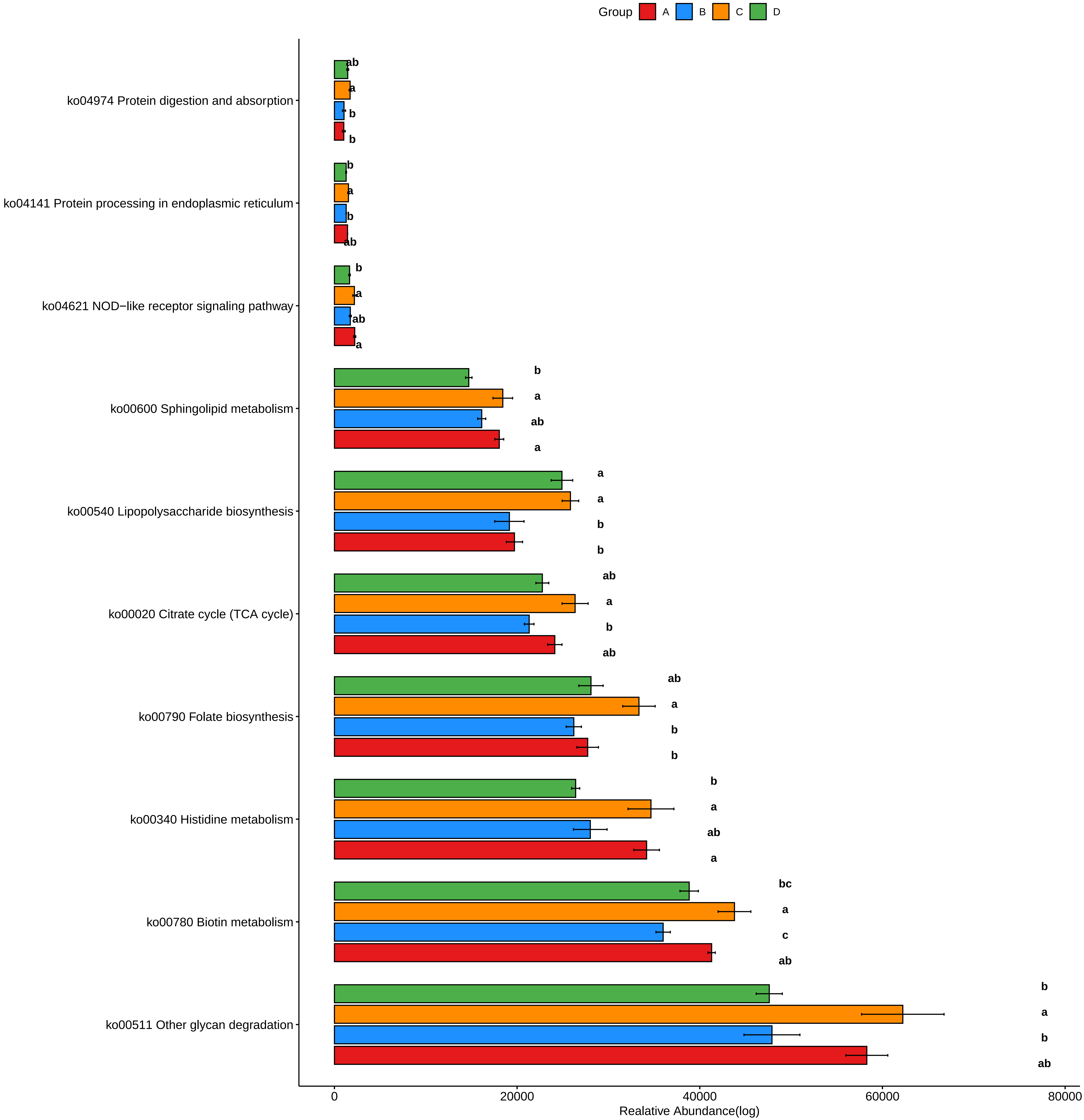
Group
A
B
C
D
ko04974 Protein digestion and absorption
ko04141 Protein processing in endoplasmic reticulum
ko04621 NOD−like receptor signaling pathway
ko00600 Sphingolipid metabolism
ko00540 Lipopolysaccharide biosynthesis
ko00020 Citrate cycle (TCA cycle)
ko00790 Folate biosynthesis
ko00340 Histidine metabolism
ko00780 Biotin metabolism
ko00511 Other glycan degradation
ab
a
b
b
b
a
b
ab
b
a
ab
a
b
a
ab
a
a
a
b
b
ab
a
b
ab
ab
a
b
b
b
a
ab
a
bc
a
c
ab
b
a
b
ab
0
20000
40000
60000
80000
Realative Abundance(log)
